# Linear trends and seasonality of births and perinatal outcomes in Upper East Region, Ghana from 2010 to 2014

**DOI:** 10.1186/s12884-016-0835-x

**Published:** 2016-03-04

**Authors:** Eric Osei, Isaac Agbemefle, Gideon Kye-Duodu, Fred Newton Binka

**Affiliations:** Department of Population and Behavioural Sciences, School of Public Health, University of Health and Allied Sciences, Ho, Volta Region Ghana; Department of Family and Community Health, School of Public Health, University of Health and Allied Sciences, Ho, Volta Region Ghana; Department of Epidemiology and Biostatistics, School of Public Health, University of Health and Allied Sciences, Ho, Volta Region Ghana; Chancellery, University of Health and Allied Sciences, Ho, Volta Region Ghana; School of Public Health, University of Health and Allied Sciences, PMB 31, Ho, Volta Region Ghana

**Keywords:** Births, Seasonality, Perinatal outcomes, Low birth weight, Stillbirth, Caesarean section

## Abstract

**Background:**

Seasonal variations greatly influence birth patterns differently from country to country. In Ghana, there is paucity of information on birth seasonal patterns. This retrospective study described the trends and seasonality of births and perinatal outcomes in Upper East Region of Ghana.

**Methods:**

Births occurring in each month of the calendar years (2010–2014; inclusive) were extracted from the District Health Information Management System (DHIMS2) database of the Bolgatanga Municipal Health Directorate and exported into Microsoft Excel spread sheet and Epi Ifo for analysis. Analysis was carried out by calculating average number of births per month correcting for unequal month length using 30 days. A Chi-square test for trend was performed to check for statistical significance (*p* < 0.05) in trends and seasonality of birth and perinatal outcomes.

**Results:**

There were 24,171 health facility deliveries, of which 97.7 % were singleton deliveries and 2.3 % were multiple (two or three) deliveries. There was a consistent rise in the annual health facility deliveries controlled for the number of fertile women, from 4169 in 2010 to 5474 in 2014 (*p* < 0.0001). Monthly birth distribution displayed a periodic pattern with peaks in May, September and October and troughs during the months of January, February and July (*p* < 0.0001). Women were likely to give birth during the raining season than the dry season. Caesarean Section (CS) rate showed a steady rise over the years (124 per 1000 births in 2010 to 185 per 1000 births in 2014 (*p* < 0.0001) with overall rate of 14.6 %. Stillbirth (SB) rate, however decreased slightly over the years from 29 per 1000 births to 23 per 1000 births (*p* = 0.197) with overall SB rate of 2.6 %. Similarly, Low Birth Weight (LBW) declined from 77 per 1000 live births to 71 per 1000 live births from 2010 to 2014 (*p* < 0.0001). Seasonal (rainy and dry) distributions did not show a clear difference in birth frequencies.

**Conclusion:**

Health facility delivery was persistently high in the Bolgatanga Municipality with birth peaking in May, September and October. Despite the rising rate of caesarean section, stillbirth rate did not significantly improved over the years. A prospective study may reveal the reasons for the increasing caesarean section rate. Additionally, understanding the factors that affect the decreasing trends of low birth weight in the municipality is crucial to public health policy makers in Ghana.

## Background

A very important feature of natality is its seasonal patterns analogous to occurrence of incidence of infections [1]. Seasonality patterns in frequency of births have been well described by many researchers [2–5] with great variation from country to country. Studies in the United States found that there is a persistent birth peak in autumn (August-September) and minimum birth in spring (March-May) [6, 7]. In Europe, major early spring peak (April) with a minor autumn peak has been described, similar to the Japanese type [8, 9]. It was reported in Australia that there was a significant shift from a September peak in early 1960s to a February-March peak in the late 1970s and also noted that there was a February-March peak in the northernmost states and a September-October peak in the southernmost States [10]. Evolving changes in the birth rhythm pattern have also been reported. In most cases, there is a common trend towards a decrease in spring births and increase in autumn births, with a decline in amplitude and subsequent loss of seasonality [9].

In Sub-Saharan Africa, the magnitude of birth outcomes changes drastically over time even within a country. A very high degree of birth seasonality was reported in Northwest Kenya as compared to other parts of Kenya [11]. Similar birth patterns were reported in four locations in Tanzania whist several locations had erratic birth patterns [12]. In Democratic Republic of Congo, birth seasonality differed among farmers [13]. Racial differences have also been documented in South Africa: birth seasonality more pronounced in blacks than whites [6]. Generally, the putative hypotheses for the variations in birth seasonality are multi-factorial in origin but can be broadly grouped into three categories: (1) seasonality due to social factors (marriage, holidays, contraception, etc.) that influence the frequency of intercourse; (2) seasonality due to climatological factors that directly affect human fecundity; and (3) seasonality due to energetic factors that principally affect female fecundity [14].

Birth outcomes are important early indicators of individual and community well-being. Of particular importance are birth weight and perinatal death, with adverse outcomes such as low birthweight (LBW), stillbirth (SB), and premature delivery presenting common threats to a child’s development, increasing a child’s risk for a variety of conditions, such as cognitive impairment and attention-deficit disorder [15]. There is paucity of evidence on birth patterns in Ghana. The aim of this study was to examine the trends and seasonality of birth frequency and perinatal outcomes in Bolgatanga Municipality of Upper East Region, Ghana.

## Methods

### Study design and setting

This was a retrospective cross sectional study of all births recorded in the Bolgatanga Municipal Health Directorate database from January, 2010 to December, 2014. The Municipality is located in the central part of the Upper East Region of Ghana, and is also the Regional Capital. It serves a population of 137,979 [16]. There are 28 public health facilities made up of 1 hospital, four health centres, five clinics and 18 Community-based Health Planning and Services (CHPS) centres, of which 26 conduct delivery services and report to the Health Directorate. There are on average 12 health facility deliveries in the Municipality every day. The climate of the municipality is classified as tropical and has two distinct seasons – a rainy season that runs from May to October and a dry season that stretches from November to April; with erratic rainfall patterns [17].

### Data source

The District Health Information Management System (DHIMS2) database is free, internet-based open source software that is being used by a number of countries all over the world and can be accessed by users who have been approved by the health facility at www.ghsdhims2.org. The database serves as a data collection, recording and compilation tool, and houses routine clinical and public health data including morbidities and mortalities, admissions, and reproductive and child health data. Data entry is done monthly at the service delivery point in list of data elements or in customized user defined forms based on paper form and are aggregated into a district summary. Data are then validated by the district health management team by running validation rules to identify violations.

### Data handling and analysis

Births occurring in each month of the calendar years during 2010–2014 (5-years) were extracted from the DHIMS2 database and exported into Microsoft Excel spread sheet and Epi Info Version 7 for analysis. Data was cleaned by detecting outliers. Outliers were detected by running analysis on the entire dataset and eliminating those points which lie far outside the general distribution and then repeating the analysis on the remaining data. The cleaned data was aggregated on monthly bases for each of the years under study. Monthly frequency of births were standardized to a common month length (30 days) using the equation: (N/d) x 30; where N is the total number of births in a given month during the entire 5-year time period and d is the number of days in that month to normalise the unequal length of each month and the existence of leap years [3]. Analysis was carried out using the standard figures to calculate the average births per calendar month for the period under study. Frequency of birth in the rainy and dry seasons was compared graphically. Trend of Traditional Birth Attendant (TBA) deliveries was compared graphically with skilled deliveries due to differences in their qualification. Chi square for trend and Odds Ratios (OR) were generated using Epi Info Version 7 and statistical significance was set at *p*-value <0.05.

In this study, low birth weight (LBW) was defined as a birth weight of a liveborn infant of less than 2.5 kg regardless of gestational age [18]. Stillbirths (SB) are babies born after 28 weeks of gestation [18]. Fresh stillbirths are babies born dead not showing signs of life at birth and no signs of maceration [18]. Traditional Birth Attendant also known as lay midwife, was defined as one who provide basic pregnancy and birthing care and advice before, during and after pregnancy and child birth based primarily on experience and knowledge acquired informally through the traditions and practices of the communities where they originated [19]. On the other hand, a skilled birth attendant was defined as “someone trained to proficiency in the skills needed to manage normal (uncomplicated) pregnancies, childbirth and the immediate postnatal period, and in the identification, management and referral of complications in women and newborns” [20]. Facility delivery was defined as any delivery occurring in an accredited healthcare facility [18].

### Ethical statement

This study was based on routine service delivery data analysis for purposes of programme monitoring and evaluation. The data analysed had no identifiers for any of the women served and was exempted from ethical approval. However, permission to analyse the data was sought from the management of the Bolgatanga Municipal Health Directorate.

## Results

There were 24,171 health facility deliveries during the study period with an average annual and monthly delivery of 4834 and 413 respectively. Overall, 97.7 % were singleton deliveries and the remaining were multiple (two or three) deliveries as shown in Table 1. Most (76.7 %) of the mothers were between the ages of 20 and 34 years old, more than 14 % were teenagers and a further 9 % were above 35 years of age. The vast majority of deliveries (84.7 %) were spontaneous vaginal deliveries and the remaining (14.5 %) were by caesarean section and vacuum (0.7 %). About 51 % of the live births were males as shown in Table 1.

**Table 1:** Obstetric characteristics of women who delivered at a health facility in Bolgatanga Municipality, Ghana, from 2010 to 2014

	Years					
Variable	2010	2011	2012	2013	2014	All years
	(*N* = 4,169)	(*N* = 4,608)	(*N* = 4,968)	(*N* = 4,952)	(*N* = 5,474)	(*N* = 24,171)
	n (%)	n (%)	n (%)	n (%)	n (%)	n (%)
Maternal Age group (years)^b^						
<20	548 (13.1)	698 (15.1)	779 (19.4)	709 (14.3)	717 (13.1)	3,451 (14.2)
20–34	3,322 (79.7)	3,451 (69.9)	3,768 (70.1)	3,806 (76.9)	4,210 (76.9)	18,557 (76.7)
35+	311(7.5)	459 (11.9)	421 (10.5)	438 (8.8)	567 (10.4)	2,196 (9.1)
Type of delivery^c^						
SVD^a^	3,631(87.2)	3,942 (85.5)	4,428 (89.1)	4,060 (82.0)	4,414 (80.6)	20,475 (84.7)
Caesarean section	519 (12.5)	637(13.8)	500 (10.1)	845 (17.1)	1,011(18.5)	3,512 (14.5)
Vacuum extraction	16 (0.4)	29 (0.6)	40 (0.8)	47 (0.9)	49 (0.9)	181 (0.7)
Sex of live babies						
Male	1,990 (47.9)	2,448 (52.3)	2,484 (50.7)	2,586 (52.2)	2,745 (50.2)	12,253 (50.8)
Female	2,167 (52.1)	2,217 (47.7)	2,412 (49.3)	2,369 (47.8)	2,725 (49.8)	11,890 (49.2)
Multiplicity of births						
Singleton (1)	4,063 (97.7)	4,533 (97.2)	4,797 (98.0)	4,853 (97.9)	5,352 (97.8)	23,626 (97.7)
Multiple (2 or 3)	94 (2.3)	132 (2.8)	99 (2.0)	102 (2.1)	118 (2.2)	545 (2.3)

^a^ *SVD* Spontaneous Vaginal Delivery; Overestimated value for maternal age^b^; 33(0.14 %), missing value for type of delivery^c^; 3 (0.01 %)

### Trends of deliveries

**Fig. 1:**
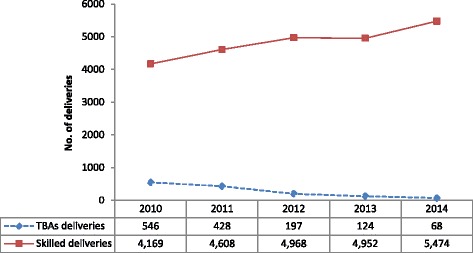
Annual trends of deliveries in Bolgatanga Municipality

Overall, delivery in a health facility was 84 % (95 % CI: 83.6–84.4). There was a steady and substantial increase in the number of health facility deliveries in the study setting, from 4169 (68 %; 95 % CI: 60–76) (monthly average: 348; range: 245–535) in the year 2010 to 5474 (99 %; (95 % CI: 96–102) (monthly average: 456; range: 340–570) in 2014 (*p* < 0.0001) with an average annual percent increase of 10 %. This observed trend was however, the opposite for TBA deliveries. During the study period, there were 1363 TBAs deliveries, which showed a progressive decline over the years from 546 in 2010 to 68 in 2014 (*p* < 0.0001); a decline rate of 87.5 % from 2010 to 2014 (Fig. 1).

### Seasonal pattern of births

**Fig. 2:**
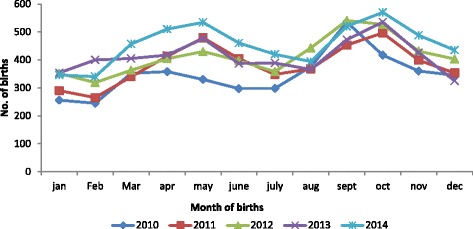
Monthly distribution of births by year in Bolgatanga Municipality

**Fig. 3:**
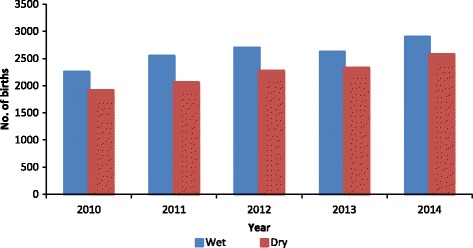
Annual seasonal birth distribution in Bolgatanga Municipality

The average monthly distribution of health facility deliveries showed a slightly periodic pattern, the peak spanning during the months of May, September and October in all of the five calendar years. On the contrary, nadir was noticed twice: during the months of February, and thereafter in July (Table 2 and Fig. 2). Seasonal distributions throughout the period under review showed higher birth rates in the rainy season as compared to the dry season (Fig. 3).

**Table 2:** Average number of births per month, 2010–2014

Month	^a^Avg. No, of births	Percentage	Odds ratio	95 % CI
January	310	6.5	1	reference
February	336	7.0	1.09	0.98–1.17
March	371	7.8	1.21	1.14–1.30
April	420	8.8	1.39	1.24–1.48
May	435	9.1	1.44	1.36–1.49
June	389	8.2	1.23	1.18–1.29
July	351	7.4	1.14	1.06–1.21
August	376	7.9	1.23	1.16–1.29
September	504	10.6	1.70	1.59–1.78
October	493	10.3	1.66	1.58–1.72
November	421	8.8	1.49	1.38–1.57
December	360	7.6	1.25	1.19–1.32
Total	4,766	100.0		

χ^2^ for trend: 33.3; *p*-value: <0.0001; ^a^corrected for unequal month length using 30 days

### Trends of perinatal outcomes

Caesarean Section rate (CS) showed a steady increase over the years from 124 per 1000 live births in 2010 (95 % CI: 113–135) to 185 per 1000 live births in 2014 (95 % CI: 74 %–196 %; *p* < 0.0001; Table 2). The overall CS rate for the study period was 14.6 % (95%CI: 14.1 %–15.0 %). Stillbirth however, declined over the years from 31 per 1000 live births in 2010 (95 % CI: 26–37) to 23 per 1000 live births in 2014 (95 % CI: 19–27) (*p* = 0.197). On the other hand, Low birthweight rate significantly declined from 144 per 1000 live births in 2010 (95 % CI: 134–155) to 74 per 1000 live births in 2014 (95 % CI: 68–82) (*p* < 0.0001) as shown in Table 3. The overall prevalence of Low birthweight and stillbirths were 10 % (95%CI: 9 %–10 %) and 3 % (95%CI: 2 %–3 %) respectively. Nearly four in ten stillbirths were fresh stillbirths.

**Table 3:** Annual trends of health facility delivery and perinatal outcomes in Bolgatanga Municipality, Ghana from 2010 to 2014

Year	Expected^a^ deliveries	HF deliveries	Percent of HF delivery	OR	95 % CI	*p*-value*
2010	6,167	4,169	68	1	Reference	<0.0001
2011	6,241	4,608	74	2.18	1.97–2.24	
2012	5,389	4,968	92	5.41	4.89–6.01	
2013	5,453	4,952	91	4.76	4.04–5.34	
2014	5,519	5,474	99	46.59	45.45–47.76	
All years	28,769	24,171	84			
Year	Total birth	SB	SB/1000 births			
2010	4,169	129	31	1	Reference	0.197
2011	4,608	77	17	0.53	0.43–1.58	
2012	4,968	177	36	1.16	1.12–1.24	
2013	4,952	115	23	0.75	0.69–0.82	
2014	5,474	126	23	0.74	0.68–0.81	
Total	24,171	619	26			
Year	Live Births	LBW	LBW/1000 live births			
2010	4,040	582	144	1	Reference	<0.0001
2011	4,531	278	61	0.39	0.31–0. 43	
2012	4,791	462	96	0.63	0.59–0.68	
2013	4,837	425	88	0.57	0.52–0.61	
2014	5,348	398	74	0.48	0.41–0.52	
Total	22,324	2,145	96			
Year	Births	CS	CS rate/1000 births			
2010	4,169	519	124	1	Reference	<0.0001
2011	4,608	637	138	1.13	1.05–1.21	
2012	4,968	500	101	0.79	0.68–0.87	
2013	4,952	845	171	1.45	1.37–1.54	
2014	5,474	1,011	185	1.60	1.52–1.68	
Total	24,171	3,512	145			

*HF* health facility *SB* Stillbirth *LBW* Low Birth Weight *CS* Caesarean section *OR* Odds Ratio *CI* Confidence Interval; *χ^2^ for trend; ^a^Expected deliveries = number of women in reproductive age

## Discussion

### Delivery trends

The single most essential intervention for reducing morbidity and mortality is to ensure that a health worker with midwifery skills is present at every child birth [21]. Considering this fact, the proportion of births attended by a skilled health personnel is used as one of the major indicators to monitor progress towards the achievement of the Millennium Development Goal of reducing maternal mortality ratio [22, 23]. We found in this study that there was a persistent increase in the proportion of births attended by skilled personnel in a health facility, ranging from 68 % in 2010 to 99 % in 2014 with the overall prevalence of 84 % during the 5 year time frame. This result corroborates with what is contained in the 2014 Ghana demographic and health survey (GDHS) report, where the proportion of births occurring in a health facility increased progressively from 42 % in 1988 to 73.1 % in 2014. In the Upper East Region, 84.1 % of births occurred in a health facility [24]. The closeness of the results lends credibility to our study findings. Dzakpasu et al., also reported a significant increase in health facility delivery over time in the Brong Ahafo Region, Ghana [25].

Our finding, however, is in contrast to findings observed in other African studies. Gitimu and colleagues [26] reported in a study in Kenya that only 40.3 % of births were attended by skilled personnel. Meselech et al. found in a qualitative study in South Central Ethiopia that majority of women gave birth at home and home delivery is taken as a common practice [27]. This difference could be attributed to the differences in financial access to maternal health services. In an attempt to increase skilled birth attendance and reduce inequality in use of services, the Government of Ghana in September 2003, introduced a policy exempting women in its four poorest regions including the region of the study site from paying for delivery services. Subsequently, in July 2008, the government introduced a policy exempting pregnant women from paying the National Health Insurance Scheme (NHIS) registration and premium fees when funding for the delivery fee exemption policy was running out [28]. These policies may have accounted for the high utilization of health facility deliveries. On the contrary, births conducted by TBAs expectedly reduced substantially from 546 births in 2010 to 68 births in 2014 representing about 87 % reduction. This downward trend observed in the TBAs deliveries could be as a result of Ghana Health Services decision of fading away TBAs deliveries due to the risk it places on the lives of both mother and child as a result of poor delivery conditions. For this reason TBAs are encourage and are given incentives to refer all labouring women to the nearest health facility and therefore the trends of deliveries conducted by them are expected to decline [17]. Meselech et al., however concluded in their study that women relied on Traditional Birth Attendants for delivery as a result of varying reasons including cost of service [27].

### Seasonal patterns of births

Using birth data from January 2010 to December 2014, this study was able to identify a strong birth peak in May, September and October and a nadir in January, February and July. Also, this study observed significant variations in average monthly frequency of births and this is in agreement with reports from other parts of the world. In India, the maximum number of births occurred in August to October and minimum in the month of January [3, 29]. Odegard observed a September peak in Norway and hypothesised that this could be due to maximum conception during the traditional mid-winter festivities [30]. Eriksson et al. [31] reported two peaks of birth in March/April and another in September/October in their record based study in Finland for the period 1650 to 1950. The National vital statistics report of the United States, indicates that births peak generally in August and reach a minimum in February [32]. However, the findings of this study contradict what was observed in other African countries. In rural Senegal, spikes of birth were observed for the month of February through May [33]. Another recent Nigerian hospital based study showed slightly sinusoidal pattern of birth with two peaks: a major peak spanning through April and May and another in October. Minimum number of births occurred in the months of July, August and December [34].

The pattern of birth seasonality exhibited by human populations around the world possibly stems from several factors, with the most important factors varying between populations and through time. Three groups of these factors have been proposed as playing important roles. These are, social factors affecting the frequency of intercourse; climatological factors affecting fecundity; and energetic factors principally affecting female ovarian function [14]. The seasonal variation in births observed in our study suggests that the women in the study site were more likely to get pregnant in the months of December/January (dry season) with births occurring in September/October (rainy season) and less likely to get pregnant in the months of April/May (rainy season) with births occurring in January/February (dry season).

Environmental conditions such as temperature, sunlight and humidity have been associated with birthing and birth outcomes [35, 36]. The dry season also referred to as the hot season in Ghana is characterised by high temperature, increased duration of sunlight and low humidity. The present study reports low birthing in the dry season as compared to the wet season. This may relate to the proportion of women birthing in healthcare facilities during the dry season. Access to healthcare facilities in the dry season may be affected by high temperature and increased duration of sunlight. In northern Ghana, women have to travel long distances (about 8 km) in order to access healthcare facilities. The heat and stress of walking long distances in the sun may encourage the patronage of substandard health services such as the Traditional birth attendance (TBA) which are within close radius to the women.

Additionally, prolonged exposures to high ambient temperatures may induce dehydration which has implications for poor pregnancy outcomes. Data from Nepal showed that the highest preterm birthing corresponded to high temperature exposures [37], possibly due to greater risk of labour induction as a result of decreased uterine blood flow when dehydration sets in [38]. On the other hand, the low birthing during the dry season can be attributed to increased risk of infection such as malaria during the rainy season. During the raining season, pregnant women in malaria endemic areas like Ghana become at higher risk of contracting malaria infection that leading to poor pregnancy outcomes such as foetal loss [36].

The seasonality patterns examined in this study may improve our understanding of the environmental factors associated with birthing and birth outcomes. The variation in the monthly frequency of births described may also have implications for public health policies and programmes on the optimal timing of interventions in the Northern part of the country aimed at improving birth outcomes and child health. A surge in a local infant population is likely to subtly influence the pattern of outbreaks of childhood diseases. When many babies are born around the same time, they become susceptible to a disease simultaneously and might thus transmit the disease more readily. Health managers may use this knowledge to plan targeted interventions to stop any possible disease outbreak.

### Trends of perinatal outcomes

According to the World Health Organization (WHO), it is unjustified to have CS rate above 15 % [39]. The observed overall CS rate for the study period was 14.6 %, close to the upper limit of the WHO’s recommended rate. The 2007 Ghana Maternal Health Survey reported CS rate of 12 % in the country and 5.1 % in the Upper East Region where this study was located. The Regional hospital being the only referral centre in the Upper East Region where this study was located receives high risk obstetric referrals from across the region and beyond, which inevitably led to increased caesarean deliveries, thus explaining our much higher proportion of caesarean sections compared to the regional estimate. Our finding is however, lower than several other studies [40–42].

The overall prevalence of stillbirths observed in this study was 26 per 1000 births, which compares favourably with reported rates in other hospital-based studies in Ghana [43–45] and other Sub-Saharan African studies [46–49]. The previous Ghanaian studies were conducted in two tertiary hospitals (Korle-Bu and Okomfo Anokye Teaching Hospitals), and being the major referral centres in the country, the hospitals receive high risk obstetric referrals which predictably result in increased adverse pregnancy outcomes, thus explaining the discrepancies in still birth rates reported in the aforementioned studies in Ghana and in this present study. The still birth prevalence reported here is consistent with the national estimate of 21 per 1000 births [50]. About 40 % of the observed stillbirths were fresh, an indication that a number of these cases could likely have been prevented [51].

According to the World Health Organization (WHO), any baby with a birth weight below 2500 g is considered to have low birthweight [52]. This study also demonstrated that LBW among live babies decreased significantly between 2010 and 2014. The decline was more pronounced from 2010 to 2011. The overall prevalence of LBW in this study was 10 %. This is slightly lower than LBW estimates from Ethiopia (11.2 %) [47]. Similarly, this finding is far lower than reports from other Ethiopian studies [53, 54]. This high discrepancy could mainly be due to methodological variations. The aforementioned studies were limited to tertiary hospital data. However, data from this present study were aggregated mainly from secondary hospitals, health centres and clinics where relatively most normal deliveries take place.

One limitation of this study is that it is hospital-based, and therefore, failed to capture all the births in the community. Thus results should be taken with some caution. Nevertheless, this is unlikely to influence the outcome of this study as over 80 % of deliveries were hospital-based.

## Conclusion

Health facility delivery is persistently high in the Bolgatanga Municipality and the birth seasonality peaked in May, September and October. Despite the rising rate of caesarean section, stillbirth rate did not significantly improved over the years. A prospective study may reveal the reasons for the increasing caesarean section rate. Moreover, understanding the factors that affect the decreasing trends of low birthweight in the municipality is crucial to public health policy makers in Ghana. Improving quality of obstetric care during labour and delivery can further reduce these adverse birth outcomes.
